# Supplementary material for: Metabolites Concentration in Plasma and Heart Tissue in Relation to High Sensitive Cardiac Troponin T Level in Septic Shock Pigs
Source: Metabolites. 2022 Apr 2;12(4):319. doi: 10.3390/metabo12040319 (PMC9024493; doi:10.3390/metabo12040319)
Supplement: Supplementary file 1 [file metabolites-12-00319-s001.zip › supplemental material.pdf]

Supplemental Material

Metabolites concentration in plasma and heart tissue in relation to high sensitive cardiac troponin T level in septic shock pigs.

Bernardo Bollen Pinto[1,2,*],Manuela Ferrario[3,*], Antoine Herpain[4,5,*], Laura Brunelli[6], Karim Bendjelid[1,7], Marta Carrara[3], Roberta Pastorelli[6].

[1]Department of Acute Medicine, Geneva University Hospitals, Geneva, Switzerland

[2]Geneva Perioperative Basic, Translational and Clinical Research Group, Geneva University Hospitals, Geneva, Switzerland

[3]Department of Electronics, Information and Bioengineering, Politecnico di Milano, Milan, Italy

[4]Department of Intensive Care, Erasme University Hospital, Université Libre de Bruxelles, Brussels, Belgium

[5]Experimental Laboratory of Intensive Care, Erasme University Hospital, Université Libre de Bruxelles, Brussels, Belgium

[6]Laboratory of Mass Spectrometry, Istituto di Ricerche Farmacologiche Mario Negri IRCCS, Milan, Italy

[7]Geneva Hemodynamic Research Group, Geneva University, Geneva, Switzerland

Corresponding author: manuela.ferrario@polimi.it

*equally contributed to the work

Table S1. List of the measurable metabolites using the Biocrates Absolute IDQ p180 platform.
Aa, acyl-acyl; ae, acyl-alkyl; a, lyso; Cx:y, where x is the number of carbons in the fatty acid side chain; y is the number of double bonds in the fatty acid side chain; DC, decarboxyl; M methyl; OH, hydroxyl; PC, phosphatidylcholine; SM, sphingomyeline

METABOLITE CLASS	#	METABOLITE NAME OR ABBREVIATION	BIOLOGICAL RELEVANCE (SELECTED EXAMPLES)
**AMINO ACIDS**	21	Alanine, arginine, aspartate, citrulline, glutamine, glutamate, glycine, histidine, isoleucine, leucine, lysine, methionine, ornithine, phenylalanine, proline, serine, threonine, tryptophan, tyrosine, valine	Amino acid metabolism, urea cycle, activity of gluconeogenesis and glycolysis, insulin sensitivity, neurotransmitter metabolism, oxidative stress
**CARNITINE**	1	C0	
**ACYLCARNITINE**	39	C2, C3, C3:1, C3-OH, C4, C4:1, C4-OH, C5, C5:1, C5:1-DC, C5-DC, C5-M-DC, C5-OH, C6, C6:1, C7-DC, C8, C9, C10, C10:1, C10:2, C12, C12-DC, C14, C14:1, C14:1-OH, C14:2, C14:2-OH, C16, C16:1, C16:1-OH, C16:2, C16:2-OH, C16-OH, C18, C18:1, C18:1-OH, C18:2	Energy metabolism, fatty acid transport and mitochondrial fatty acid oxidation, ketosis, oxidative stress, mitochondrial membrane damage
**BIOGENIC AMINES**	19	Acetylornithine, asymmetric dimethylarginine, total dimethylarginine, alpha-aminoadipic acid, carnosine, creatinine, histamine, kynurenine, methionine sulfoxide, nitrotyrosine, hydroxyproline, phenylethylamine, putrescine, sarcosine, serotonin, spermidine, spermine, taurine	Neurological disorders, cell proliferation, cell cycle progression, DNA stability, oxidative stress
**LYSO-PHOSPHATIDYLCHOLINES**	14	lysoPC a C14:0 / C16:0/ C16:1/ C17:0/ C18:0/ C18:1/ C18:2/ C20:3/ C20:4/ C26:0/ C26:1/ C28:0/ C28:1	Degradation of phospholipids, membrane damage, signaling cascades, fatty acid profile
**DIACYL-PHOSPHATIDYLCHOLINES**	38	PC aa C24:0/ C26:0/ C28:1/ C30:0/ C30:2/ C32:0/ C32:1/ C32:2/ C32:3/ C34:1/ C32:2/ C34:3/ C32:4/ C36:0/ C36:1/ C36:2/ C36:3/ C36:4/ C36:5/ C36:6/ C38:0/ C38:1/ C38:3/ C38:4/ C38:5/ C38:6/ C40:1/ C40:2/ C40:3/ C40:4/ C40:5/ C40:6/ C42:0/ C42:1/ C42:2/ C42:4/ C42:5/ C42:6	
**ACYL-ALKYL-PHOSPHATIDYLCHOLINE**	38	PC ae C30:0/ C30:2/ C32:1/ C32:2/ C34:0/ C34:1/ C34:2/ C34:3/ C36:0/ C36:1/ C36:2/ C36:3/C36:4/C36:5/C38:0/C38:1/C38:2/C38:3/C38:4/C38:5/C38:6/C40:1/C40:2/ C40:3/ C40:4/C40:5/C40:6/C42:0/C42:1/C42:2/C42:3/C42:4/C42:5/C44:3/C44:4/C44:5/ C44:6	Dyslipidemia, membrane composition and damage, fatty acid profile, activity of desaturases
**SPHINGOMYELINS**	15	SM (OH) C14:1, SM C16:0, SM C16:1, SM C16:1, SM C18:0, SM C18:1, SM C20:2, SM C22:3, SM (OH) C22:1, SM (OH) C22:2, SM C24:0, SM C24:1, SM (OH) C24:1, SM C26:0, SM C26:1	Signaling cascades, membrane damage (eg. neurodegeneration)
**HEXOSE**	1	H1	Carbohydrate metabolism
**TOTAL**	186		

List of abbreviations used: Ala, Alanine; Arg, Arginine; Asn, Asparagine; Asp, Aspartate; Cit, Citrulline; Gln, Glutamine; Glu, Glutamate; Gly, Glycine; His, Histidine; Ile, Isoleucine; Leu, Leucine; Lys, Lysine; Met, Methionine; Orn, Ornithine; Phe, Phenylalanine; Pro, Proline; Ser, Serine; Thr , Threonine Trp, Tryptophan; Tyr, Tyrosine; Val, Valine; Ac-Orn, Acetylornithine; total DMA, total dimethylarginine; ADMA, Asymmetric dimethylarginine; SDMA, Symmetric dimethylarginine; alpha-AAA, alpha-Aminoadipic acid; Met-SO, Methioninesulfoxide; Nitro-Tyr, Nitrotyrosine; c4-OH-Pro, cis-4-Hydroxyproline; t4-OH-Pro, trans-4Hydroxyproline; PEA , Phenylethylamine

Table S2 - The level of all the measured metabolites in the animal experiment (plasma and heart tissue) organized in a excel table..

Table S3 – Clinical and laboratory data at each time point of the experiment. Values are reported as median (25th,75th) percentile for the septic animal only (n=3 low hs-cTnT, n=3 high hs-cTnT).

	low hs-cTnT			high hs-cTnT		
	**Baseline**	**Shock**	**Full resuscitation**	**Baseline**	**Shock**	**Full resuscitation**
		n=3			n=3	
**hs-cTnT (ng/L)**	15.00 (12.75, 19.50)	21.00 (16.50, 35.25)	26.00 (26.00, 29.00)	8.00 (8.00, 9.50)	21.00 (18.00, 24.00)	48.00 (42.00, 58.50)
**Lactate mmol/L**	0.90 (0.90, 0.90)	1.30 (1.22, 1.75)	1.90 (1.45, 1.90)	0.90 (0.90, 0.90)	2.10 (1.65, 2.55)	3.10 (3.02, 3.55)
**HR bpm**	75.00 (72.75, 97.50)	129.00 (104.25, 130.50)	144.00 (141.00, 150.00)	82.00 (65.50, 88.75)	160.00 (115.75, 162.25)	150.00 (145.50, 152.25)
**DAP rad mmHg**	55.00 (54.25, 60.25)	37.00 (35.50, 40.00)	48.00 (43.50, 51.75)	57.00 (54.75, 58.50)	33.00 (31.50, 36.00)	35.00 (34.25, 40.25)
**MAP rad mmHg**	71.00 (71.00, 76.25)	46.00 (45.25, 47.50)	70.00 (65.50, 75.25)	76.00 (73.75, 77.50)	46.00 (45.25, 46.00)	55.00 (51.25, 64.00)
**CO ml/min**	4700 (4400, 5075)	2800 (1900, 2950)	8000 (7550, 8825)	5400 (4500, 5775)	4850 (3700, 6000)	9900 (9675, 11100)
**SvO2 %**	63.00 (61.50, 68.25)	53.00 (50.75, 54.50)	78.00 (74.25, 80.25)	67.00 (61.75, 68.50)	68.00 (65.00, 69.50)	68.00 (64.25, 74.75)
**T °C**	38.80 (38.50, 39.10)	39.20 (38.45, 39.72)	39.00 (37.95, 39.08)	39.00 (38.85, 39.08)	37.70 (37.70, 38.30)	38.50 (38.28, 39.10)
**pH**	7.48 (7.47, 7.49)	7.41 (7.38, 7.43)	7.45 (7.42, 7.45)	7.48 (7.47, 7.49)	7.42 (7.41, 7.46)	7.43 (7.35, 7.44)
**$PaCO_2$ mmHg**	46.70 (45.58, 49.47)	48.70 (47.42, 49.52)	47.60 (45.05, 48.73)	45.60 (45.15, 47.48)	48.90 (45.07, 49.95)	48.80 (48.05, 56.15)
**PaO_2 mmHg**	124 (122, 131)	143 (136, 152)	157 (115, 163)	129 (126, 137)	134 (94, 149)	70 (63, 90)
**HCO_3^- mmol/L**	33.20 (33.05, 36.80)	30.70 (27.93, 30.93)	30.00 (30.00, 32.25)	33.00 (32.25, 34.95)	31.20 (31.20, 31.35)	31.40 (30.20, 31.70)
**BE**	8.90 (8.83, 11.90)	5.20 (2.35, 5.73)	5.20 (4.75, 7.53)	8.40 (8.10, 10.35)	5.90 (5.82, 6.80)	6.10 (3.85, 6.78)
**Sat O2 %**	100.00 (99.25, 100.00)	100.00 (99.25, 100.00)	99.00 (98.25, 99.75)	100.00 (99.25, 100.00)	100.00 (97.00, 100.00)	89.00 (88.25, 95.75)
**Hct %**	28.40 (25.85, 29.60)	33.00 (29.25, 36.00)	27.00 (24.75, 29.25)	26.00 (24.50, 27.50)	34.00 (26.50, 38.50)	24.20 (21.05, 30.80)
**Na+ mmol/L**	133 (130, 137)	131 (128, 136)	134 (133, 135)	132 (131, 134)	131 (131, 133)	133 (132, 134)
**K+ mmol/L**	4.30 (3.78, 4.38)	4.30 (3.78, 4.45)	4.40 (4.25, 4.78)	4.00 (4.00, 4.00)	3.80 (3.50, 3.80)	4.30 (4.23, 4.52)
**Cl- mmol/L**	100.00 (97.75, 100.75)	99.00 (96.00, 100.50)	98.00 (97.25, 99.50)	99.00 (97.50, 102.00)	97.00 (96.25, 98.50)	97.00 (97.00, 98.50)
**anion gap**	-1.20 (-16.42, -0.30)	5.00 (2.22, 5.00)	6.00 (-12.30, 6.00)	-1.00 (-1.00, -0.70)	2.80 (1.30, 5.65)	6.20 (2.45, 6.50)
**Ca++ mmol/L**	1.26 (1.24, 1.28)	1.21 (1.19, 1.28)	1.10 (1.08, 1.14)	1.20 (1.15, 1.22)	1.21 (1.13, 1.25)	1.17 (1.03, 1.19)
**Glucose mmol/L**	86.00 (77.75, 100.25)	71.00 (68.00, 79.25)	86.00 (62.00, 104.75)	94.00 (92.50, 97.75)	62.00 (61.25, 77.75)	64.00 (61.00, 68.50)

Table S4 – Metabolites concentration in heart tissue in the two groups (LV, left ventricle; RV, right ventricle; RA, right atria). The two groups consist in the animals with a low or high hs-cTnT concentration at the end of the experiment. Values are reported as median (25th,75th) percentile. The concentrations are reported as μM.
Wilcoxon ranksum test: *p<0.05 low vs high hs-cTnT.

	**LV**		**RV**		**RA**	
	**low**	**high**	**low**	**high**	**low**	**high**
**lysoPC a C16:0**	0.43 (0.33, 0.59)	0.33 (0.32, 0.54)	0.40 (0.29, 0.68)	0.46 (0.36, 1.33)	0.52 (0.39, 0.57)	0.35 (0.28, 0.38)
**lysoPC a C18:0**	0.20 (0.17, 0.36)	0.16 (0.15, 0.24)	0.25 (0.17, 0.38)	0.25 (0.22, 0.52)	0.31 (0.22, 0.45)	0.18 (0.17, 0.28)
**lysoPC a C18:1**	1.02 (0.75, 1.11)	0.58 (0.55, 0.65)	0.77 (0.52, 0.93)	0.61 (0.57, 0.62)	0.75 (0.71, 0.90)	0.56 (0.46, 0.65)
**lysoPC a C18:2**	2.26 (1.51, 2.99) *	1.08 (0.94, 1.29)	1.94 (1.42, 2.76)	1.01 (0.97, 1.81)	0.77 (0.65, 0.88)	0.44 (0.44, 0.61)
**lysoPC a C20:3**	0.14 (0.10, 0.18)	0.08 (0.06, 0.08)	0.13 (0.10, 0.18) *	0.07 (0.07, 0.09)	0.08 (0.05, 0.09)	0.05 (0.05, 0.07)
**lysoPC a C20:4**	1.71 (1.47, 2.51) *	0.70 (0.69, 1.13)	1.54 (1.19, 1.85)	0.78 (0.72, 0.85)	1.15 (0.93, 1.69)	0.78 (0.77, 1.12)
**lysoPC a C26:1**	0.09 (0.06, 0.13) *	0.04 (0.04, 0.05)	0.07 (0.04, 0.12)	0.04 (0.03, 0.06)	0.05 (0.04, 0.07)	0.04 (0.03, 0.05)
**lysoPC a C28:1**	0.03 (0.03, 0.04) *	0.01 (0.01, 0.02)	0.03 (0.02, 0.04)	0.01 (0.01, 0.02)	0.03 (0.03, 0.06)	0.02 (0.01, 0.05)
**PC aa C30:0**	0.12 (0.10, 0.15)	0.10 (0.09, 0.13)	0.12 (0.12, 0.13)	0.11 (0.10, 0.16)	0.17 (0.10, 0.23)	0.14 (0.10, 0.15)
**PC aa C30:2**	0.02 (0.02, 0.03) *	0.01 (0.01, 0.02)	0.02 (0.02, 0.03)	0.02 (0.01, 0.03)	0.04 (0.03, 0.04)	0.03 (0.02, 0.04)
**PC aa C32:0**	12 (8, 19)	10 (9, 10)	10 (7, 15)	11 (7, 17)	21 (7, 28)	12 (6, 15)
**PC aa C32:1**	7.79 (6.39, 9.43)	6.72 (4.98, 6.86)	5.36 (4.76, 7.46)	6.72 (5.53, 10.53)	12.10 (8.01, 15.10)	11.10 (10.95, 11.63)
**PC aa C32:2**	3.43 (2.83, 3.62)	3.59 (2.65, 3.63)	2.79 (2.18, 3.02)	3.63 (2.78, 4.47)	3.11 (1.79, 3.52)	3.18 (3.04, 3.73)
**PC aa C32:3**	1.59 (1.45, 1.75)	1.19 (1.04, 1.62)	1.28 (1.18, 1.40)	2.10 (1.32, 2.40)	0.82 (0.44, 1.02)	0.75 (0.73, 0.83)
**PC aa C34:1**	104 (84, 144)	107 (76, 111)	74 (55, 77)	89 (61, 141)	164 (80, 179)	115 (82, 122)
**PC aa C34:2**	144 (113, 176)	117 (92, 148)	129 (104, 142)	174 (118, 224)	100 (53, 132)	79 (77, 88)
**PC aa C34:3**	13 (11, 17)	11 (10, 13)	11 (10, 13)	15 (10, 18)	12 (8, 14)	13 (10, 13)
**PC aa C34:4**	1.99 (1.66, 2.28)	1.64 (1.59, 1.81)	1.44 (1.26, 2.07)	2.16 (1.84, 2.60)	1.87 (1.42, 2.46)	2.30 (2.05, 2.64)

**PC aa C36:0**	8.38 (5.99, 9.15)	8.34 (6.46, 8.43)	4.65 (4.29, 4.74)	* 5.99 (5.68, 9.90)	3.66 (1.94, 4.00)	4.96 (3.29, 5.84)
**PC aa C36:1**	4.91 (3.48, 6.38)	4.19 (3.79, 7.42)	4.18 (3.77, 5.02)	5.46 (4.25, 6.44)	8.29 (5.75, 14.00)	4.70 (3.69, 9.05)
**PC aa C36:2**	22 (19, 32)	20 (18, 28)	28 (22, 30)	* 39 (34, 49)	37 (23, 44)	28 (27, 37)
**PC aa C36:3**	21 (16, 26)	17 (15, 19)	19 (16, 24)	28 (24, 30)	37 (24, 42)	40 (31, 43)
**PC aa C36:4**	37 (24, 44)	21 (20, 28)	27 (19, 32)	32 (29, 33)	46 (28, 53)	38 (38, 41)
**PC aa C36:5**	4.70 (3.98, 6.73)	3.87 (3.44, 3.88)	3.97 (2.98, 4.42)	4.66 (3.87, 6.62)	6.04 (5.26, 7.81)	6.20 (5.36, 6.25)
**PC aa C36:6**	0.79 (0.73, 0.91)	* 0.58 (0.58, 0.61)	0.55 (0.50, 0.77)	0.62 (0.49, 1.24)	0.59 (0.53, 0.76)	0.55 (0.35, 0.66)
**PC aa C38:0**	1.17 (0.83, 1.28)	1.01 (0.91, 1.10)	0.80 (0.56, 0.99)	0.80 (0.78, 1.60)	1.06 (0.29, 1.28)	1.02 (0.77, 1.21)
**PC aa C38:1**	0.69 (0.58, 0.79)	0.83 (0.71, 0.88)	0.48 (0.42, 0.50)	0.45 (0.31, 0.94)	0.44 (0.17, 0.47)	* 0.49 (0.48, 0.52)
**PC aa C38:3**	0.85 (0.56, 1.21)	0.59 (0.58, 0.73)	0.62 (0.57, 0.73)	* 1.35 (1.14, 1.81)	1.29 (0.88, 2.12)	1.45 (0.77, 1.58)
**PC aa C38:4**	4.41 (3.52, 7.01)	3.71 (3.21, 5.77)	4.87 (4.34, 5.84)	* 7.06 (6.80, 8.45)	10.65 (8.42, 15.10)	9.02 (8.03, 10.73)
**PC aa C38:5**	4.55 (3.65, 6.53)	2.91 (2.72, 3.90)	3.87 (3.04, 4.50)	4.91 (4.83, 4.93)	11.25 (7.75, 13.30)	10.30 (8.93, 11.20)
**PC aa C38:6**	2.72 (1.83, 3.02)	1.48 (1.41, 1.91)	2.17 (1.59, 2.99)	2.43 (2.29, 2.96)	4.92 (3.96, 6.94)	4.52 (4.36, 5.65)
**PC aa C40:3**	0.07 (0.05, 0.09)	0.06 (0.05, 0.06)	0.08 (0.07, 0.11)	0.07 (0.02, 0.09)	0.08 (0.07, 0.12)	0.09 (0.03, 0.10)
**PC aa C40:4**	0.15 (0.13, 0.19)	* 0.07 (0.04, 0.07)	0.09 (0.05, 0.13)	0.17 (0.11, 0.23)	0.33 (0.15, 0.43)	0.20 (0.17, 0.33)
**PC aa C40:5**	0.24 (0.20, 0.43)	0.34 (0.23, 0.35)	0.31 (0.24, 0.44)	0.44 (0.38, 0.51)	0.95 (0.41, 1.36)	0.58 (0.35, 0.77)
**PC aa C40:6**	0.51 (0.40, 0.55)	0.41 (0.28, 0.44)	0.48 (0.43, 0.56)	0.48 (0.46, 0.74)	1.38 (0.75, 1.66)	0.97 (0.84, 1.23)
**PC aa C42:1**	0.03 (0.01, 0.05)	0.01 (0.00, 0.01)	0.01 (0.00, 0.01)	0.01 (0.01, 0.01)	0.03 (0.00, 0.04)	0.01 (0.00, 0.02)
**PC aa C42:5**	0.04 (0.02, 0.06)	0.02 (0.01, 0.02)	0.02 (0.01, 0.03)	0.04 (0.02, 0.05)	0.02 (0.01, 0.04)	0.01 (0.01, 0.02)
**PC ae C30:1**	0.08 (0.06, 0.10)	* 0.03 (0.03, 0.04)	0.07 (0.05, 0.09)	* 0.03 (0.02, 0.04)	0.05 (0.03, 0.11)	0.03 (0.02, 0.08)
**PC ae C30:2**	0.02 (0.01, 0.02)	0.01 (0.01, 0.02)	0.02 (0.02, 0.03)	0.01 (0.01, 0.02)	0.02 (0.01, 0.04)	0.01 (0.01, 0.03)
**PC ae C32:1**	2.48 (1.85, 3.05)	2.43 (1.66, 2.62)	1.71 (1.48, 1.98)	2.71 (1.68, 3.33)	3.71 (2.22, 4.53)	2.60 (2.03, 3.27)
**PC ae C32:2**	2.28 (1.74, 2.57)	1.80 (1.61, 2.03)	1.47 (1.42, 1.49)	2.25 (1.53, 2.97)	2.04 (1.71, 2.36)	2.20 (2.03, 2.25)
**PC ae C34:1**	8.14 (5.73, 13.90)	9.14 (7.02, 11.21)	5.91 (4.64, 6.79)	8.04 (5.32, 15.51)	14.75 (8.48, 19.10)	12.10 (10.32, 12.47)
**PC ae C34:2**	69 (42, 101)	77 (49, 87)	47 (42, 50)	72 (44, 101)	46 (25, 49)	42 (31, 42)
**PC ae C34:3**	138 (97, 189)	164 (106, 189)	99 (84, 123)	193 (103, 243)	35 (22, 44)	32 (23, 39)
**PC ae C36:1**	1.41 (1.26, 1.54)	1.60 (1.07, 1.63)	1.21 (1.08, 1.30)	1.58 (1.42, 1.91)	1.91 (1.29, 2.33)	1.62 (1.03, 1.79)

**PC ae C36:2**	3.80 (2.94, 5.57)	4.19 (2.95, 4.66)	2.79 (2.33, 3.05)	4.18 (3.16, 6.56)	4.38 (2.81, 4.85)	4.24 (3.36, 4.32)
**PC ae C36:3**	16 (9, 21)	19 (13, 22)	10 (9, 13)	19 (13, 24)	11 (6, 13)	8 (7, 10)
**PC ae C36:4**	45 (32, 51)	40 (35, 45)	31 (28, 38)	52 (36, 69)	34 (26, 45)	40 (30, 44)
**PC ae C36:5**	108 (81, 130)	96 (87, 105)	61 (58, 79)	74 (71, 147)	65 (36, 77)	74 (42, 83)
**PC ae C38:0**	0.35 (0.31, 0.48)	0.43 (0.30, 0.44)	0.31 (0.27, 0.40)	0.35 (0.33, 0.37)	0.54 (0.38, 0.63)	0.58 (0.51, 0.64)
**PC ae C38:1**	0.21 (0.17, 0.25)	0.16 (0.08, 0.23)	0.13 (0.09, 0.16) *	0.34 (0.33, 0.45)	0.13 (0.10, 0.30)	0.19 (0.12, 0.26)
**PC ae C38:2**	0.25 (0.20, 0.28)	0.15 (0.08, 0.19)	0.22 (0.16, 0.23)	0.21 (0.18, 0.30)	0.23 (0.18, 0.39)	0.27 (0.27, 0.32)
**PC ae C38:3**	0.32 (0.31, 0.41)	0.31 (0.28, 0.38)	0.30 (0.19, 0.41)	0.48 (0.36, 0.52)	0.59 (0.41, 0.69)	0.54 (0.46, 0.75)
**PC ae C38:4**	1.41 (0.91, 1.61)	1.20 (1.06, 1.21)	1.19 (0.87, 1.28)	1.51 (1.45, 2.18)	2.92 (2.34, 3.16)	2.68 (2.47, 2.75)
**PC ae C38:5**	11 (9, 14)	9 (9, 11)	7 (6, 7) *	10 (9, 16)	19 (11, 21)	16 (14, 20)
**PC ae C38:6**	25 (17, 28)	21 (19, 22)	15 (13, 16)	19 (14, 32)	12 (6, 14)	13 (8, 16)
**PC ae C40:1**	0.20 (0.17, 0.38)	0.10 (0.09, 0.16)	0.23 (0.17, 0.26)	0.23 (0.11, 0.26)	0.57 (0.49, 0.66)	0.58 (0.44, 0.85)
**PC ae C40:2**	0.07 (0.04, 0.09)	0.05 (0.05, 0.08)	0.08 (0.04, 0.12)	0.07 (0.06, 0.10)	0.12 (0.08, 0.17)	0.08 (0.07, 0.10)
**PC ae C40:5**	0.27 (0.23, 0.29) *	0.14 (0.13, 0.15)	0.18 (0.15, 0.20)	0.28 (0.23, 0.35)	0.44 (0.34, 0.77)	0.60 (0.48, 0.60)
**PC ae C40:6**	0.65 (0.53, 0.79)	0.60 (0.58, 0.62)	0.49 (0.45, 0.55)	0.60 (0.53, 1.13)	1.25 (0.61, 1.44)	0.98 (0.90, 1.22)
**PC ae C44:6**	0.05 (0.03, 0.06)	0.03 (0.02, 0.03)	0.01 (0.00, 0.06)	0.02 (0.01, 0.05)	0.03 (0.01, 0.04)	0.00 (0.00, 0.02)
**Ac-Orn**	1.54 (1.05, 2.22)	2.71 (1.59, 2.86)	1.38 (0.79, 2.32)	2.77 (1.83, 2.87)	0.74 (0.48, 1.05)	0.83 (0.66, 1.53)
**alpha-AAA**	0.71 (0.24, 1.92)	1.24 (0.75, 1.61)	0.77 (0.12, 1.22)	1.21 (0.77, 1.44)	2.10 (1.99, 3.51)	2.26 (1.42, 4.73)
**Carnosine**	1.59 (1.43, 5.05)	2.61 (2.55, 3.07)	3.09 (1.97, 6.35)	5.35 (5.31, 5.39)	4.14 (3.37, 4.75)	7.20 (4.79, 7.39)
**Putrescine**	0.97 (0.85, 1.20)	1.52 (1.18, 3.15)	1.10 (0.54, 1.27)	2.76 (1.98, 3.76)	0.43 (0.15, 0.56)	0.34 (0.28, 1.40)
**Spermidine**	0.51 (0.25, 0.81)	0.43 (0.34, 0.69)	0.57 (0.45, 0.62)	0.79 (0.48, 0.83)	0.21 (0.14, 0.41)	0.44 (0.20, 0.49)
**Ala**	226 (180, 369)	332 (293, 436)	208 (148, 264)	442 (408, 560)	176 (124, 239)	246 (235, 482)
**Arg**	9.20 (5.90, 12.50)	9.65 (7.89, 12.99)	8.07 (6.05, 9.65)	9.65 (9.58, 10.81)	5.46 (4.03, 7.45)	5.75 (5.53, 9.09)
**Asn**	9 (8, 21)	17 (12, 19)	10 (4, 15)	23 (17, 25)	14 (8, 15)	8 (7, 13)
**Asp**	20 (12, 31)	24 (17, 32)	22 (15, 30)	29 (21, 30)	40 (26, 49)	32 (29, 34)
**Cit**	19 (8, 29)	24 (18, 24)	8 (4, 13)	14 (9, 19)	4 (4, 8)	7 (7, 9)
**Gln**	985 (555, 1410)	1485 (1095, 1575)	778 (580, 940)	1050 (949, 1181)	205 (182, 245)	306 (173, 542)
**Glu**	125 (72, 151)	96 (63, 97)	94 (78, 113)	73 (71, 120)	146 (122, 190)	136 (127, 156)

**Gly**	39 (32, 45)	80 (78, 100)	50 (35, 55)	* 102 (101, 109)	54 (52, 62)	* 84 (83, 106)
**Ile**	6.55 (3.53, 7.35)	7.50 (6.90, 8.10)	6.50 (4.68, 7.65)	8.60 (7.62, 8.71)	6.97 (4.80, 8.00)	6.20 (6.20, 7.44)
**Lys**	15 (7, 21)	21 (16, 21)	13 (11, 15)	19 (16, 19)	9 (7, 12)	8 (8, 13)
**Phe**	5.78 (3.86, 6.85)	8.65 (7.07, 9.25)	5.54 (4.00, 6.45)	8.60 (8.22, 9.95)	5.13 (3.59, 6.00)	6.40 (5.99, 8.50)
**Pro**	11 (9, 14)	* 21 (20, 28)	11 (9, 13)	* 27 (27, 27)	13 (11, 14)	* 26 (22, 34)
**Ser**	8 (4, 17)	14 (14, 19)	6 (5, 17)	18 (13, 20)	12 (12, 13)	16 (14, 18)
**Thr**	13 (5, 14)	17 (15, 17)	9 (8, 16)	19 (14, 25)	14 (13, 17)	20 (16, 22)
**Val**	10 (5, 14)	14 (11, 15)	10 (8, 13)	13 (13, 15)	10 (8, 14)	11 (11, 14)
**SM (OH) C16:1**	0.02 (0.01, 0.03)	0.02 (0.01, 0.03)	0.02 (0.02, 0.03)	0.02 (0.01, 0.02)	0.02 (0.02, 0.03)	0.01 (0.01, 0.02)
**SM (OH) C22:1**	0.001 (0.001, 0.003)	0.003 (0.003, 0.004)	0.002 (0.001, 0.003)	0.004 (0.001, 0.005)	0.002 (0.000, 0.003)	0.001 (0.001, 0.004)
**SM (OH) C22:2**	0.001 (0.001, 0.001)	0.002 (0.001, 0.003)	0.001 (0.001, 0.001)	* 0.005 (0.004, 0.008)	0.001 (0.001, 0.002)	0.001 (0.001, 0.002)
**SM (OH) C24:1**	0.002 (0.001, 0.002)	0.001 (0.001, 0.001)	0.001 (0.001, 0.001)	0.002 (0.001, 0.002)	0.001 (0.001, 0.001)	0.001 (0.001, 0.002)
**SM C16:0**	0.93 (0.54, 1.13)	0.86 (0.53, 0.87)	0.76 (0.71, 0.92)	0.95 (0.87, 0.99)	1.04 (0.61, 1.21)	0.78 (0.66, 0.97)
**SM C16:1**	0.06 (0.05, 0.06)	0.06 (0.05, 0.06)	0.05 (0.05, 0.06)	0.07 (0.07, 0.09)	0.11 (0.07, 0.12)	0.11 (0.11, 0.11)
**SM C18:0**	0.26 (0.17, 0.36)	0.26 (0.19, 0.29)	0.21 (0.17, 0.29)	0.25 (0.17, 0.27)	0.25 (0.14, 0.33)	0.19 (0.11, 0.26)
**SM C18:1**	0.05 (0.05, 0.06)	0.06 (0.04, 0.07)	0.05 (0.03, 0.06)	0.06 (0.06, 0.06)	0.10 (0.08, 0.11)	0.10 (0.09, 0.11)
**SM C24:0**	0.007 (0.006, 0.008)	0.005 (0.004, 0.009)	0.007 (0.004, 0.008)	0.012 (0.008, 0.015)	0.007 (0.006, 0.010)	0.007 (0.005, 0.010)
**SM C24:1**	0.01 (0.01, 0.01)	0.02 (0.01, 0.02)	0.01 (0.01, 0.01)	0.01 (0.01, 0.03)	0.01 (0.01, 0.01)	0.00 (0.00, 0.01)
**C0**	18 (16, 19)	24 (19, 27)	15 (13, 21)	22 (20, 27)	13 (10, 21)	16 (13, 29)
**C18**	0.50 (0.10, 1.53)	1.14 (0.37, 1.57)	0.55 (0.10, 1.11)	0.52 (0.35, 1.07)	0.51 (0.13, 0.52)	0.23 (0.23, 0.72)
**C18:1**	0.28 (0.06, 1.18)	1.12 (0.33, 1.21)	0.37 (0.09, 0.84)	0.39 (0.26, 0.57)	0.35 (0.07, 0.42)	0.17 (0.12, 0.71)
**C2**	6.96 (1.87, 8.59)	4.87 (2.79, 6.70)	4.05 (1.65, 6.19)	5.03 (4.67, 7.99)	3.29 (1.19, 4.19)	3.69 (2.63, 4.89)
**C3-DC (C4-OH)**	1.25 (0.47, 1.68)	0.86 (0.34, 1.23)	0.86 (0.53, 1.37)	1.20 (0.94, 1.36)	0.51 (0.14, 0.89)	0.49 (0.32, 1.17)
**C4**	0.82 (0.26, 0.97)	0.76 (0.38, 0.83)	0.52 (0.30, 0.77)	0.84 (0.51, 1.06)	0.33 (0.15, 0.37)	0.31 (0.24, 0.47)

Table S5- VIP scores of PLS-DA obtained from 36 features, which consist in the metabolites fold change between the values after full resuscitation and baseline in the animal study.
122 metabolites were measured and detected in peripheral blood at each time point. We considered the sum of lysophosphadylcoline 16 and 18 (lysoPC16+18), the sum of all the sphingomyelin (SM), the sum of all the phosphadylcoline (PC), the amino acids, biogenic amines and sugars for a total of 36 features. We computed the fold change Res./BL for these features. We performed a partial least square discriminant analysis (PLS-DA) by using 3 latent variables in order to separate the two animal groups, i.e. low vs high hs-cTnT. The features with a VIP score>1 are shaded in grey.

Features	VIP score
sugars	1.95
t4-OH-Pro	1.69
Pro	1.47
Creatinine	1.41
SM tot	1.24
Gly	1.22
Taurine	1.21
PC	1.20
Gln	1.16
Ala	1.14
Trp	1.09
Cit	1.04
Leu	1.01
Lys	0.97
Carnosine	0.96
Orn	0.95
Ser	0.95
ADMA	0.94
Thr	0.92
Serotonin	0.87
lysoPC16+18	0.84
alpha-AAA	0.84
total DMA	0.83
Asn	0.83
Arg	0.75
His	0.75
Putrescine	0.71
Glu	0.69
C2	0.67
Tyr	0.65
Ac-Orn	0.64
Phe	0.54
Ile	0.51
Met	0.43
Asp	0.43
Val	0.11

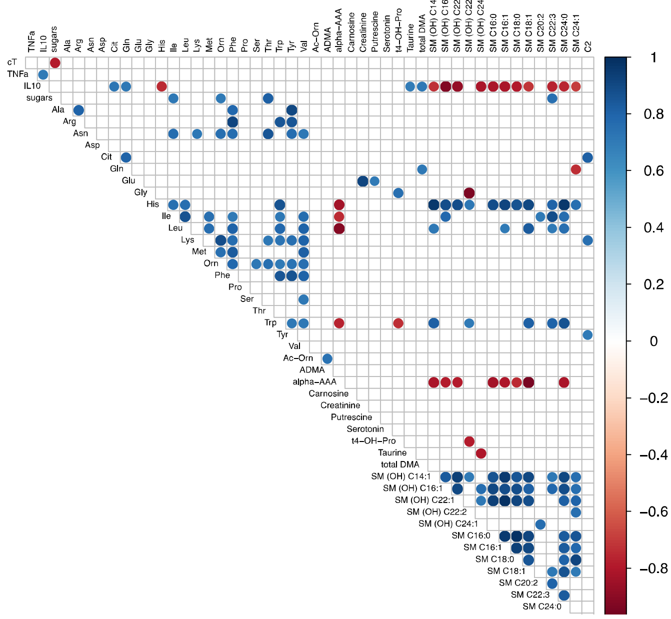

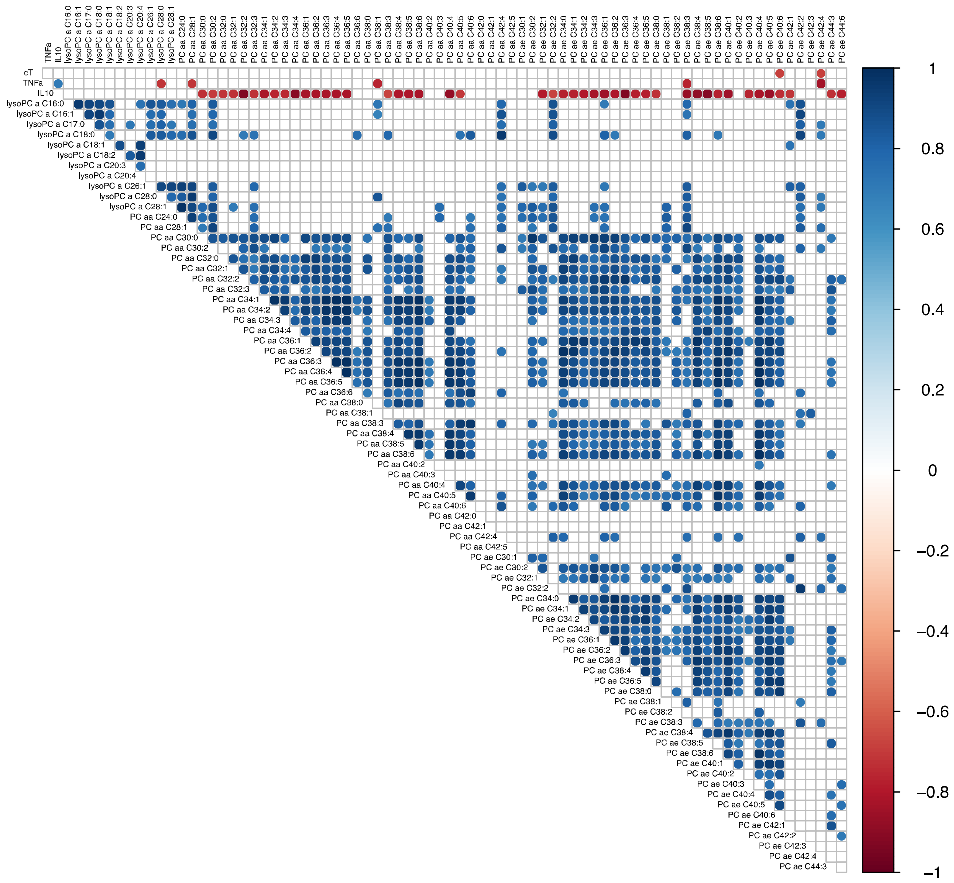

Figure S1 -Correlation matrices representing the correlation coefficients between the fold change in concentrations of hs-cTnT (cT), cytokines (TNF-alpha and IL-10) and metabolites evaluated in n=9 pigs. The fold change represent the ratio between the values measured after development of septic shock and baseline (SS/BL). Only the significant correlation are reported (Spearman p-value< 0.5). The red dots represents negative correlations, whereas blue dots positive ones. The correlations with cTn can be observed in the first row.
